# Bipolar and monopolar transurethral resection of the prostate are equally effective and safe in this high quality randomized controled trial

**DOI:** 10.1590/S1677-5538.IBJU.2019.0766.1

**Published:** 2020-11-18

**Authors:** Cristiano M. Gomes, Julyana K. M. Moromizato, Thulio B. V. Brandão

**Affiliations:** 1 Universidade de São Paulo Faculdade de Medicina Departamento de Cirurgia São PauloSP Brasil Divisão de Urologia do Departamento de Cirurgia da Faculdade de Medicina da Universidade de São Paulo, São Paulo, SP, Brasil

## COMMENT

Numerous surgical techniques are approved for the surgical treatment of benign prostatic obstruction (BPO). They include minimally invasive procedures such as the newly introduced prostatic urethral lift and water vapor thermal therapy, transurethral resection, vaporization or enucleation of the prostate and open or laparoscopic/robotic assisted prostatectomy and have been recommended by the guidelines of the most distinguished scientific organizations (1, 2). In clinical practice for many decades, transurethral resection of the prostate (TURP) remains the standard by which subsequent surgical modalities for the treatment of BPH have been compared.

Guidelines recommend that either monopolar or bipolar TURP may be used, for patients with a moderately enlarged prostate, of up to 80 cc, depending on the surgical expertise of the practitioner (1, 2). In bipolar TURP (B-TURP), the energy does not travel through the body to reach a skin pad, as is the case for monopolar TURP (M-TURP). It is confined between the active and passive poles situated on the resectoscope tip (resection loop) (3). It may be performed in 0.9% NaCl solution and does not require the use of isoosmolar solutions (mannitol, glycine), greatly reducing the risk for acute dilutional hyponatremia and the TUR syndrome. This is especially important for larger prostates requiring prolonged surgery (4).

Many studies have been published in recent years exploring the use of B-TURP and comparing it with M-TURP. Systematic reviews have also compared the two techniques, confirming comparable efficacy for both and a reduced risk for acute dilutional hyponatremia and TUR syndrome for B-TURP (5, 6). Although some studies indicate a reduced risk for blood transfusion and clot retention with B-TURP, the evidence is not strong to make a recommendation in this regard (2, 7).

There are different bipolar resection devices and no evidence in favor of a specific system (3). In the present study, Otaola-Arca H. et al. (8) used the Plasma KineticTMSuperpulse generator as the energy source for bipolar TURP (PK-TURP) and prospectively compared it with M-TURP. They included patients with refractory LUTS and/or complications associated with BPO and a prostate volume < 80 cc. Of 100 randomized patients, 84 were included in the final analysis. Patients were evaluated after 1, 3, 6 and 12 months and the efficacy variables were improvement in the International Prostate Symptom Score (IPSS), quality of life question of the IPSS, Qmax, postvoid residue, prostate volume and sexual function measured by the IIEF-5. The authors showed comparable efficacy and safety outcomes for the two methods. The only difference observed was a greater improvement of the QoL score in patients who underwent PK-TURP, which was minor and considered clinically insignificant. The efficacy results of this study are in accordance with a recent meta-analysis by Cornu et al. that showed no differences comparing the two techniques (9). However, the meta-analysis showed an increased risk for dilutional hyponatremia and bleeding complications (clot urinary retention and transfusion rate) with M-TURP (9).

An important aspect of the present study was the strict methodological criteria adopted. Based on the Jadad scale that assess the methodological quality of randomized control trials, most previous studies comparing PK-TURP with M-TURP had one or more methodological issues (10). The present study has a very high methodological design based on the Jadad score (score 4/5). However, it has the limitation of providing a relatively short follow-up of one year. Few studies provided long-term results such as those from Al-Rawashdah et al. (11) and Xie, et al. (12), who followed the patients for at least 3 years and showed comparable results in the long-term (Table-1). As recommended by Cornu et al. (9) “Further studies are needed to provide long-term comparative data and head-to head comparisons” and we can only hope that the authors will continue to follow these patients regularly and report on the long-term results.

**Table 1:** Efficacy and safety of PK-TURP vs M-TURP in RCTs.

Series	Patients (N)	Follow up (months)	Efficacy	Safety
Otaola-Arca et al, 2020 (8)	84	12	NS*	NS
de Sio et al., 2006 (14)	70	12	NS	NS
Seckiner et al., 2006 (15)	48	12	NS	More hyponatremia in M-TURP
Nuhoglu et al., 2006 (16)	54	12	NS	More hyponatremia in M-TURP
Yoon et al., 2006 (17)	102	12	NS	NS
Eturhan et al., 2007 (18)	240	12	Greater Qmax improvement with PK-TURP	More bleeding in M-TURP
Iori et al., 2008 (19)	51	12	NS	NS
Kong et al., 2009 (20)	102	12	NS	More hyponatremia and Hb decline in M-TURP
Bhansali et al., 2009 (21)	67	12	NS	More hyponatremia in M-TURP
Autorino et al., 2009 (22)	70	48	NS	NS
Shinghania et al., 2010 (23)	60	12	Greater Qmax improvement with PK-TURP	NS
Xie et al., 2012 (12)	220	60	NS	More hyponatremia and Hb decline in M-TURP
Giulianelli et al., 2013 (24)	160	36	NS	More Hb decline in M-TURP

* Legends: QoL question statistically different favoring PK-TURP

**RCTs**= randomized control trials; NS= significant; **IPSS**= International Prostate Symptom Score; **Qmax**= maximum flow rate; **M-TURP**= monopolar transurethral resection of the prostate; **PK-TURP**= Bipolar TURP using the Plasma Kinetic generator; **Hb**= Hemoglobulin

Efficacy parameters= Qmax, IPSS, postvoid residue.

**Safety parameters** = Bleeding (transfusion, clot retention, hemoglobin decline), TUR syndrome, hyponatremia

NOTE: not all studies evaluated all parameters.

Another potential problem that deserves attention is the fact that the study was conducted in a university hospital and surgeries were performed by practitioners with varying levels of experience. It certainly might be seen as a limitation, but the fact that it provides the outcomes of both surgical techniques in the daily practice is relevant and the fact that a sub-analysis based on the level of surgical experience did not show differences in primary and secondary outcomes is reassuring.

Finally, since cost-effectiveness studies are very important to determine the value of technologies and treatments, and guide public policies for patient management, it is a little frustrating that the authors did not look at this aspect in the study. A recent systematic review comparing M-TURP with B-TURP using a different energy source favoured the B-TURP. (13) There are no data cost-effectiveness analysis for PK-TURP and this could be assessed by the authors in future studies.

## References

[1] 1. Gravas S, Cornu JN, Gacci M, Gratzke C, Herrmann TRW, Mamoulakis C, et al. EAU Guidelines on Management of Non-Neurogenic Male Lower Urinary Tract Symptoms (LUTS), including Benign Prostatic Obstruction (BPO). European Association of Urology; 2019. [Internet] Available at. <https://uroweb.org/wp-content/uploads/EAU-Guidelines-on-the-Management-of-Nonneurogenic-Male-LUTS-2018-large-text.pdf>

[2] 2. Foster HE, Barry MJ, Dahm P, Gandhi MC, Kaplan SA, Kohler TS, et al. Surgical Management of Lower Urinary Tract Symptoms Attributed to Benign Prostatic Hyperplasia: AUA Guideline. J Urol. 2018;200:612-9. 10.1016/j.juro.2018.05.048 29775639

[3] 3. Issa MM. Technological advances in transurethral resection of the prostate: bipolar versus monopolar TURP. J Endourol. 2008;22:1587-95. 10.1089/end.2008.0192 18721041

[4] 4. Rassweiler J, Teber D, Kuntz R, Hofmann R. Complications of transurethral resection of the prostate (TURP)––incidence, management, and prevention. Eur Urol. 2006;50:969-79; discussion 980. 10.1016/j.eururo.2005.12.042 16469429

[5] 5. Mamoulakis C, Ubbink DT, de la Rosette JJ. Bipolar versus monopolar transurethral resection of the prostate: a systematic review and meta-analysis of randomized controlled trials. Eur Urol. 2009;56:798-809. 10.1016/j.eururo.2009.06.037 19595501

[6] 6. Omar MI, Lam TB, Alexander CE, Graham J, Mamoulakis C, Imamura M, et al. Systematic review and metaanalysis of the clinical effectiveness of bipolar compared with monopolar transurethral resection of the prostate (TURP). BJU Int. 2014;113:24-35. 10.1111/bju.12281 24053602

[7] 7. Tang Y, Li J, Pu C, Bai Y, Yuan H, Wei Q, et al. Bipolar transurethral resection versus monopolar transurethral resection for benign prostatic hypertrophy: a systematic review and meta-analysis. J Endourol. 2014;28:1107-14. 10.1089/end.2014.0188 PMC4146489 24754254

[8] 8. Otaola-Arca H, Álvarez-Ardura M, Molina-Escudero R, Fernández MI, Páez-Borda Á. A prospective randomized study comparing bipolar plasmakinetic transurethral resection of the prostate and monopolar transurethral resection of the prostate for the treatment of Benign Prostatic Hyperplasia: efficacy, sexual function, Quality of Life, and complications. Int Braz J Urol. 2021;47:131 44. 10.1590/S1677-5538.IBJU.2019.0766 PMC7712692 33047918

[9] 9. Cornu JN, Ahyai S, Bachmann A, de la Rosette J, Gilling P, Gratzke C, et al. A Systematic Review and Meta-analysis of Functional Outcomes and Complications Following Transurethral Procedures for Lower Urinary Tract Symptoms Resulting from Benign Prostatic Obstruction: An Update. Eur Urol. 2015;67:1066-96. 10.1016/j.eururo.2014.06.017 24972732

[10] 10. Jadad AR, Moore RA, Carroll D, Jenkinson C, Reynolds DJ, Gavaghan DJ, et al. Assessing the quality of reports of randomized clinical trials: is blinding necessary? Control Clin Trials. 1996;17:1-12. 10.1016/0197-2456(95)00134-4 8721797

[11] 11. Al-Rawashdah SF, Pastore AL, Salhi YA, Fuschi A, Petrozza V, Maurizi A, et al. Prospective randomized study comparing monopolar with bipolar transurethral resection of prostate in benign prostatic obstruction: 36-month outcomes. World J Urol. 2017;35:1595-601. 10.1007/s00345-017-2023-7 28243790

[12] 12. Xie CY, Zhu GB, Wang XH, Liu XB. Five-year follow-up results of a randomized controlled trial comparing bipolar plasmakinetic and monopolar transurethral resection of the prostate. Yonsei Med J. 2012;53:734-41. 10.3349/ymj.2012.53.4.734 PMC3381470 22665339

[13] 13. Treharne C, Crowe L, Booth D, Ihara Z. Economic Value of the Transurethral Resection in Saline System for Treatment of Benign Prostatic Hyperplasia in England and Wales: Systematic Review, Meta-analysis, and Cost-Consequence Model. Eur Urol Focus. 2018;4:270-9. 10.1016/j.euf.2016.03.002 28753756

[14] 14. de Sio M, Autorino R, Quarto G, Damiano R, Perdonà S, di Lorenzo G, et al. Gyrus bipolar versus standard monopolar transurethral resection of the prostate: a randomized prospective trial. Urology. 2006;67:69-72. 10.1016/j.urology.2005.07.033 16413335

[15] 15. Seckiner I, Yesilli C, Akduman B, Altan K, Mungan NA. A prospective randomized study for comparing bipolar plasmakinetic resection of the prostate with standard TURP. Urol Int. 2006;76:139-43. 10.1159/000090877 16493215

[16] 16. Nuhoğlu B, Ayyildiz A, Karagüzel E, Cebeci O, Germiyanoğlu C. Plasmakinetic prostate resection in the treatment of benign prostate hyperplasia: results of 1-year follow up. Int J Urol. 2006;13:21-4. 10.1111/j.1442-2042.2006.01218.x 16448427

[17] 17. Yoon CJ, Kim JY, Moon KH, Jung HC, Park TC. Transurethral resection of the prostate with a bipolar tissue management system compared to conventional monopolar resectoscope: one-year outcome. Yonsei Med J. 2006;47:715-20. 10.3349/ymj.2006.47.5.715 PMC2687758 17066516

[18] 18. Erturhan S, Erbagci A, Seckiner I, Yagci F, Ustun A. Plasmakinetic resection of the prostate versus standard transurethral resection of the prostate: a prospective randomized trial with 1-year follow-up. Prostate Cancer Prostatic Dis. 2007;10:97-100. 10.1038/sj.pcan.4500907 16926854

[19] 19. Iori F, Franco G, Leonardo C, Laurenti C, Tubaro A, D-Amico F, et al. Bipolar transurethral resection of prostate: clinical and urodynamic evaluation. Urology. 2008;71:252-5. 10.1016/j.urology.2007.09.064 18308095

[20] 20. Kong CH, Ibrahim MF, Zainuddin ZM. A prospective, randomized clinical trial comparing bipolar plasma kinetic resection of the prostate versus conventional monopolar transurethral resection of the prostate in the treatment of benign prostatic hyperplasia. Ann Saudi Med. 2009;29:429-32. 10.4103/0256-4947.57163 PMC2881428 19847078

[21] 21. Bhansali M, Patankar S, Dobhada S, Khaladkar S. Management of large (>60 g) prostate gland: PlasmaKinetic Superpulse (bipolar) versus conventional (monopolar) transurethral resection of the prostate. J Endourol. 2009;23:141-5. 10.1089/end.2007.0005 19178175

[22] 22. Autorino R, Damiano R, Di Lorenzo G, Quarto G, Perdonà S, D'Armiento M, et al. Four-year outcome of a prospective randomised trial comparing bipolar plasmakinetic and monopolar transurethral resection of the prostate. Eur Urol. 2009;55:922-9. 10.1016/j.eururo.2009.01.028 19185975

[23] 23. Singhania P, Nandini D, Sarita F, Hemant P, Hemalata I. Transurethral resection of prostate: a comparison of standard monopolar versus bipolar saline resection. Int Braz J Urol. 2010;36:183-9. 10.1590/s1677-55382010000200008 20450503

[24] 24. Giulianelli R, Albanesi L, Attisani F, Gentile BC, Vincenti G, Pisanti F, et al. Comparative randomized study on the efficaciousness of endoscopic bipolar prostate resection versus monopolar resection technique. 3 year follow-up. Arch Ital Urol Androl. 2013;85:86-91. 10.4081/aiua.2013.2.86 23820656

